# Quadcopter Flight Control Using a Non-invasive Multi-Modal Brain Computer Interface

**DOI:** 10.3389/fnbot.2019.00023

**Published:** 2019-05-31

**Authors:** Xu Duan, Songyun Xie, Xinzhou Xie, Ya Meng, Zhao Xu

**Affiliations:** School of Electronics and Information, Northwestern Polytechnical University, Xi'an, China

**Keywords:** multi-modal EEG, motor imagery, SSVEP, eye movement, quadcopter flight control

## Abstract

Brain-Computer Interfaces (BCIs) translate neuronal information into commands to control external software or hardware, which can improve the quality of life for both healthy and disabled individuals. Here, a multi-modal BCI which combines motor imagery (MI) and steady-state visual evoked potential (SSVEP) is proposed to achieve stable control of a quadcopter in three-dimensional physical space. The complete information common spatial pattern (CICSP) method is used to extract two MI features to control the quadcopter to fly left-forward and right-forward, and canonical correlation analysis (CCA) is employed to perform the SSVEP classification for rise and fall. Eye blinking is designed to switch these two modes while hovering. Real-time feedback is provided to subjects by a global camera. Two flight tasks were conducted in physical space in order to certify the reliability of the BCI system. Subjects were asked to control the quadcopter to fly forward along the zig-zag pattern to pass through a gate in the relatively simple task. For the other complex task, the quadcopter was controlled to pass through two gates successively according to an S-shaped route. The performance of the BCI system is quantified using suitable metrics and subjects are able to acquire 86.5% accuracy for the complicated flight task. It is demonstrated that the multi-modal BCI has the ability to increase the accuracy rate, reduce the task burden, and improve the performance of the BCI system in the real world.

## Introduction

Brain-computer interfaces (BCIs) have provided an entirely new communication way to interact for both disabled and healthy people with the external world. Such a BCI system is achieved through sensing, processing, and actuation (Nicolas-Alonso and Gomez-Gil, 2012). An electrophysiological signal is firstly collected, amplified, and digitized. A computer then interprets the underlying neurophysiology of the signal in order to translate user's intents into the specific commands. These commands are finally actuated by the external devices. Moreover, the user could receive feedback in order to adjust his thoughts to generate updated and adapted commands (Yuan and He, 2014). Among non-invasive BCI, the scalp-recorded electroencephalogram (EEG) is widely used due to its high temporal resolution and convenience of use. Brain patterns including event-related desynchronization/synchronization (ERD/ERS), steady-state visual evoked potential (SSVEP), and P300 potential are utilized for the EEG-based BCI (Hong and Khan, 2017). Upon performing motor imagery, local neuron populations over a sensorimotor area in charge of imagination task experience a desynchronization and result in a decrease of mu-beta power, which is accompanied by an increased synchronization in the non-task hemisphere. These phenomena are termed ERD and ERS, respectively (Pfurtscheller et al., 2006). SSVEP is a continuous oscillating response from the posterior scalp of the brain to a stimulus flickering at a constant frequency. The amplitude of SSVEP is enhanced when a subject's attention is cued to the stimulus (Xie et al., 2016). P300 is one of the components for event-related potentials (ERPs) that indicate the responses to specific cognitive, sensory, or motor events. The presentation of a stimulus in an oddball paradigm could produce a positive peak which appeared 300 ms after the onset of the stimulus (Xie et al., 2014; Ramadan and Vasilakos, 2017).

Many researchers have begun to focus on flying robot navigation in real space using BCI recently, since it could achieve flexible movement in 3D real space, and any remote mobile device which has meaningful interaction with the real world could be a substitute as well. Shi et al. investigated the Unmanned Aerial Vehicle (UAV) control by using a left/right-hand MI-based BCI and semi-autonomous navigation for indoor target searching, where MI EEG is simply employed to choose or not choose the current feasible direction (Shi et al., 2015). Kim et al. demonstrated the viability of flight control using a hybrid interface with EEG and eye tracking. Eight different directions were achieved by using eye tracking, while mental concentration detected by EEG is only utilized for switching (Kim et al., 2014). Though these flying robots are controlled with a relatively high accuracy, BCI is only responsible for a small part of the control system.

Some researchers have also investigated the brain-controlled flying robot when the BCI is applied to users to deal with all the commands. On the one hand, Audrey et al. used four-class hand motor imagery to fly a virtual helicopter in a 3-D world with the aid of intelligent control strategies (Royer et al., 2010). LaFleur et al. successfully conducted a quadcopter flight control experiment using a MI-based BCI in physical world. Separable control of three dimensions was obtained by imagining clenching of left hand, right hand, both hands, and idle state. The success rate reached 79.2% (LaFleur et al., 2013). To improve the success rate of motor imagery signals, various feature extraction algorithms such as the common spatial pattern, cross-correlation method, and neural network by complex Morlet wavelets were investigated (Shi et al., 2015; Das et al., 2016; Zhang et al., 2019). Moreover, the computational cost should be paid attention to ensure real-time operation. To this end, an improved CSP method called the complete information common spatial pattern (CICSP) is selected in our system, which employs additional intermediate spatial filters to extract more discriminable features in motor imagery. These early efforts have laid the groundwork for other research teams; however, in this scenario, individual imagining movements may generate a mental burden. Furthermore, performing a continuous mental task to control a quadcopter in real time could be an exhausting procedure, and when added to environmental distractions, it could lead to a loss of control over the quadcopter.

On the other hand, SSVEP-based evoked BCI systems could be set up easily with almost no training. Other researchers have focused on developing a SSVEP-based BCI system with a shorter time and lower error rate (Middendorf et al., 2000; Liu et al., 2018). In order to overcome the discomfort of eyes due to flickering, Wang et al. designed a wearable BCI system based on 4-class SSVEP which presented using a head-mounted device (Wang et al., 2018). Although it alleviates the user's visual burden to some extent, this was only conducted in the simulated 3D environment.

Considering these problems, a multi-modal BCI based on the combination of different brain patterns was introduced recently which is capable of beating specific targets more successfully than a single-modal BCI (Li et al., 2016). The active MI task combined with the reactive SSVEP task is commonly used to constitute the multi-modal BCI system. It reduces the mental burden, decelerating visual fatigue over time. This multi-modal BCI consists of two protocols; that is, performing both imagining movements and focusing on oscillating visual attention simultaneously as well as executing two tasks separately (Allison et al., 2010, 2012). A previous work found the dual-task interference; that is, performing a simultaneous SSVEP task might impair the performance of an ERD task, whereas performing a secondary task (such as ERD) does not impair the performance on a primary task (such as SSVEP) (Pfurtscheller et al., 2010a; Das et al., 2016). Performing two tasks separately could be more in line with the physiological basis. Therefore, in this case, few studies have investigated a protocol that performs motor imagery and visual attention separately. A study developed a sequentially operating hybrid BCI that used a one-channel imagery-based BCI to turn on/off an SSVEP BCI (Pfurtscheller et al., 2010b; Brunner et al., 2011). Horki et al. designed a hybrid BCI that employed imaging the brisk feet dorsi flexion to control the open and close function of a gripper, and focusing on flickering lights to control the extension and flexion function of the elbow (Horki et al., 2011). Duan et al. employed three SSVEP signals and one feet motor imagery signal to design a hybrid BCI system which could provide both manipulation and mobility commands to a service robot. Moreover, Alpha rhythm is considered as a switch from SSVEP to motor imagery (Duan et al., 2015). These methods described above inspired the protocol of the multi-modal BCI-controlled quadcopter in our research.

In this study, we investigate the capacity of a flying robot controlled using MI and SSVEP combined with multi-modal BCI in the three-dimensional physical world, aiming at enhancing the ability of interacting with the outside world. Subjects are trained to imagine left/right-hand movement as well as to gaze at two flickering lights which generate ERD/ERS and SSVEP in order to actuate the quadcopter in both horizontal and vertical directions, respectively. Moreover, two modes are switched by eye blinking, since people who have motor neuro disease could nonetheless perceive and respond to the external world by receiving visual stimuli and eye movements effectively (Lin et al., 2010). Control commands decoded from EEG are transmitted with a fixed time interval to the quadcopter for updating its motor direction via Wi-Fi. The real-time video acquired from the global camera is sent back to the subject's monitor in order to complete the BCI control by telepresence. Two outdoor flight experiments were performed and some metrics were utilized to evaluate the performance of the multi-modal BCI system.

The remainder of this paper is arranged as follows. In section Materials and Methods, the experimental set-up and paradigm are explained, and the methods of EEG pattern recognition are given. In addition, the metrics of performance analysis are also introduced in this section. The experimental results are depicted in section Results. Section Discussion includes the discussion. Finally, section Conclusion summarizes the conclusions obtained.

## Materials and Methods

### Architecture of BCI System

**Figure 1:**
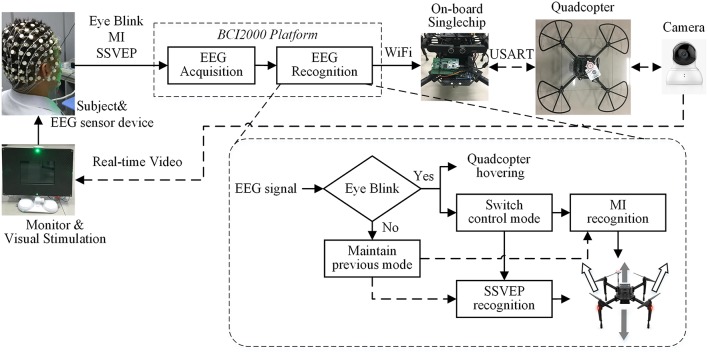
Architecture of the multi-modal BCI system for Quadcopter control.

Figure 1 shows the architecture of the multi-modal BCI system for quadcopter flight control. Subjects perform three types of tasks—motor imagery, SSVEP, and eye blinking to control the quadcopter flying in the physical environment. The EEG signals were collected from a comfortable and easy-to-use Geodesic Sensor Net (Electrical Geodesics Inc, OR) before they were imported into the Net Amps 300 amplifier (Electrical Geodesics Inc, OR) to get the low-noise and high-quality data. The EEG data acquisition and pattern recognition were run on BCI2000 platform. The EEG data was decoded into five specific patterns, that is flying left-forward, flying right-forward, rise, fall, and flight mode switch while flight hovering, as shown by means of the gray and white arrows, specifically. The decoded outputs were transmitted to the quadcopter's onboard single chip via Wi-Fi, in which they were converted into the control instructions. After that, the instructions were sent to the flight control system by USART to complete the continuous control of the quadcopter. The subject watched the monitor of the experimental site real-time video from a mounted camera; meanwhile, executed the appropriate tasks to control the quadcopter autonomously. Two flight modes, MI mode and SSVEP mode, took charge of the actuation of the horizontal and vertical dimensions, respectively, and the two modes were switched by eye blinking. As for the stimulation of SSVEP, subjects had to gaze at one of the two green LEDs which were placed on the top and bottom of the monitor, while it did not require any for MI mode.

### Experiment Layout

**Figure 2:**
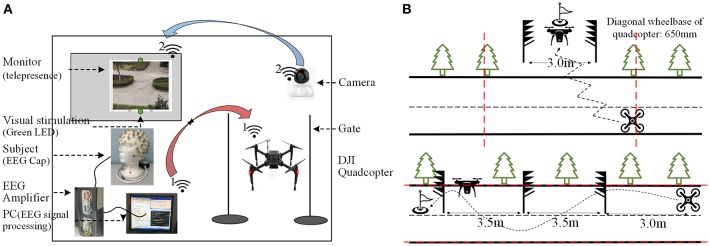
The view of the experiment layout **(A)** The sketch map of the experiment set-up. **(B)** The simpler and more complex flight tasks.

Figure 2A illustrates the experiment layout of the flight controlled by using the BCI system. The flight environment was set up on vacant land outside the laboratory building while subjects controlled the flight autonomously in a corner of the land using telepresence. Subjects were asked to sit in a comfortable chair with their arms relaxing on the chair handle. The monitor was adjusted so that the screen was exactly at the center of the subjects' visual field. The length and width of the screen were 16 and 12 cm, respectively, and the distance between the two LEDs was 23.5 cm. Outdoors, two barriers were made on one gate so that the quadcopter could pass through, and the top of the gate was ~2.5 m above the ground. The quadcopter could not fly out of the top of the gate. Subjects were situated on the back of the flight field to ensure safety. Since the subject's sight was blocked out, the flight status of the quadcopter was presented to the subject by a monitor from the camera.

In order to prove the reliability of the BCI system, the flight tasks consisted of two phases, as shown in Figure 2B. The first one was relatively simple for the subject to familiarize themselves with the real-time BCI system in the physical world, and the second task was more complex in order to testify the performance of the flight controlling system. The pre-designed trajectory of the first flight task is shown in the top (b). The quadcopter was controlled to fly forward along the zigzag pattern, while its height was adjusted so that it could pass through the gate, which was 3 meters wide and 1.5 meters high, and subsequently landed at the designated destination area. The second task is illustrated in the bottom (b). The quadcopter's starting position was located nearly on the extension of the connection line of three obstacles and three meters away from the first obstacle. Subjects were asked to control the quadcopter to pass through two gates successively according to an S-shaped route. The quadcopter bypassed the first barrier and then adjusted its flight direction to pass through the first 3.5-m-wide gate, which was immediately followed by the second direction change, and then it passed through the second gate of the same size and finally landed at the designated destination area. During the experiment, subjects were required to provide instructions for the task and destination, of which they were informed in advance. Other experimental personnel were not allowed to give any tips to the subjects. In order to ensure the security of the flight, the safety boundaries of the control area were stipulated as the vertical extension lines of the trees beside the two barriers for the simple task, as well as two edges of the road for the complex flight task (as shown by the red dotted line in Figure 2B). Once the quadcopter went beyond the boundaries, the experimenter landed the quadcopter in a safe position by using the remote controller and announced an end of this trial instantly.

The Matrix 100 (M100) quadcopter (DJI, Shenzhen, China) was chosen as the external device for the BCI experiment due to the extensive open source platform, which was suitable for scientific research, and it was able to expand the capabilities of the aerial platform with an onboard embedded system that supported serial communication as well as DJI SDK. It provided the interface to program a wide range of speed and yaw rate in three dimensions. In addition, the M100 quadcopter provided a stable and reliable flight, with up to 40 min of flight time.

### Calibration Phase

Before steering an actual quadcopter, a training phase was carried out in a virtual quadcopter flight simulator developed by DJI with the main purpose being to calibrate subjects' control signals. A target instruction consisting of left-forward, right-forward, rise, fall, as well as eye blinking were informed to the subject by sound. EEG signals were collected to train the recognition method for the three modes. The training would not stop until subjects obtained a success rate of 80% or above for three separate modals (Zhang et al., 2016).

### Experimental Paradigm

Each trial started after the quadcopter took off, hovering about 1 m off the ground. Imagining left-hand movement turned the quadcopter to the left-forward direction at an angle of −42 degrees, with the forward direction speed at 0.25 m/s, while imagining right-hand movement turned the quadcopter to right-forward direction at a symmetrical angle to the left and the same speed. Gazing at the top flickering LED for a climb with a speed of 0.2 m/s while gazing at the bottom flickering LED made the quadcopter descend with a speed of −0.3 m/s. When the system switched to MI mode, the LEDs stopped flickering and subjects had to concentrate on thinking of left- or right-hand movement continuously. Consciously blinking eyes kept the quadcopter hovering, and two flight modes switched simultaneously. The 1.5 s time window of raw EEG signal was pattern-recognized online to generate a control output which was sent wirelessly every 1 s to the quadcopter's onboard single chip.

Nine human subjects (one female and eight males, aged 22–32) were recruited to participate in this experiment. The details of the subjects are listed in Table 1. Three of them attended the BCI experiment before and the rest were naive to the experiment. All subjects had normal or corrected-to-normal visual acuity. The experimental procedures were approved by the Northwestern Polytechnical University Hospital Ethics Committee. Subjects attended the experiment for 8 non-consecutive days, with about 5 trials each session each day. Subjects were instructed to avoid body movement during each trial. The raw EEG signal was recorded from 12 electrodes (CP1, CP2, FC1, FC2, FC3, FC4, C1, C2, C3, C4, Oz, Fp2), although the Geodesic Sensor Net contained 64 electrodes of the standard 10/20 international EEG positioning system. A reference electrode Cz was placed on the central-parietal area. All impedances were kept below 5 kΩ. EEG data were band-pass filtered between 0.3 and 100 Hz and sampled at 1,000 Hz. The EEG processing was performed in MATLAB R2013a (The MathWorks, Inc., Natick, MA, USA). If the quadcopter passed through the gate navigating by the subject successfully, a “gate acquisition” was recorded; however, if the quadcopter went beyond the borders of the control area, an “out of borders” was recorded.

**Table 1:** The details of subjects.

**Subject number**	**Age**	**Sex**	**Handedness**	**Corrected visual acuity**	**Former experimental experience**
1	23	Male	Right	0.8/0.8	Yes
2	22	Female	Right	0.9/0.8	No
3	32	Male	Right	1/0.9	No
4	22	Male	Right	1/1.2	No
5	25	Male	Right	0.8/0.9	Yes
6	25	Male	Left	0.8/0.8	Yes
7	25	Male	Right	1/1	No
8	26	Male	Right	0.9/0.9	No
9	26	Male	Right	1/0.9	No

### Pattern Recognition Methods

**Figure 3:**
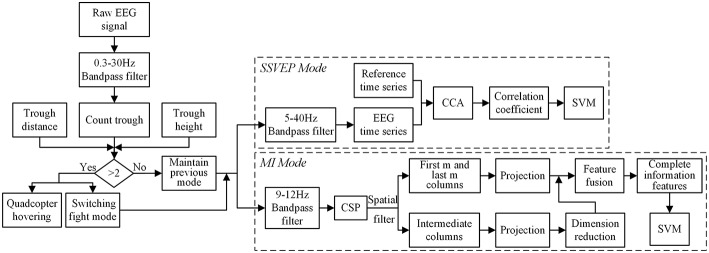
The procedure of recognition for three EEG patterns.

Figure 3 illustrates the procedure of eye blinking, SSVEP, and MI recognition algorithms. Firstly, a block of the raw EEG signal is detected by the count trough method. Once the number of troughs within a block was higher than 2, the quadcopter kept hovering, and the two flight modes switched simultaneously. Otherwise, this signal block is sent to the previous mode, which is either SSVEP or MI mode for processing.

### Eye Blinking

Eye blinking was used to switch between the two flight modes. Conscious eye blinking is defined as excessive effort blinking with a fixed frequency; that is, blinking twice within a 1.5 s EEG data block. The EEG time series of the conscious eye blinking was recorded by the Fp2 channel, which is located at the upper right orbital in the prefrontal lobe. It demonstrated that the downward vertical eye movement could generate a large-magnitude negative deflection, representing thus a remarkable trough for an eye blinking (Corby and Kopell, 1972). A counting trough method was proposed to the detect eye blinking of the subject. The flow chart of the method is shown in the left part of Figure 3. A 0.3–30 Hz bandpass filter was employed since the frequency range of eye movement activity is maximal at frequencies below 4 Hz. While removing the EEG baseline and high-frequency noise, the characteristic waveform of eye blinking was preserved. Then the number of troughs was counted in a 1.5 s time window under the constraints of the average distance between two troughs *d* and the minimum absolute height of the trough *h*. Two constraints we set here aimed at differentiating conscious eyes blinking from normal blinking or other noise like head movements or frowning. The parameter d was set as 0.75 s long, while the parameter h was averaged among the filtered EEG measurements of calibration dataset. If the number of troughs was >2, the subject was considered as eye blinking; otherwise, it was further recognized within MI/SSVEP modes.

### SSVEP

SSVEP is a periodic evoked potential induced by rapidly repetitive visual stimulation, typically at specific frequencies >6 Hz. Subjects were able to steer the quadcopter rise and fall simply by gazing at the top and bottom LED flicking at 12.4 and 18 Hz, respectively (Liu et al., 2018). The EEG signals were bandpass filtered between 5 and 40 Hz, which included the fundamental frequency of the visual stimulus and its first harmonic (Müller-Putz et al., 2005). The canonical correlation analysis (CCA) frequency recognition method, which is the most commonly used feature extraction method, was employed for SSVEP detection, as shown in the upper right part of Figure 3. CCA was able to obtain the correlation between two sets of variables in general. Considering two sets of variables ***X*** and ***Y***, CCA aims to find a pair of vectors *a* and *b*, which could maximize the correlation between *x* = *a*^*T*^*X* and *y* = *b*^*T*^*Y*. The maximization problem can be described as below:

(1) $$\begin{array}{l} \max corr\left(x,y\right)=\frac{a^{T}\mathrm{cov}\left(X,Y\right)b}{\sqrt{a^{T}\mathrm{cov}\left(X,X\right)a}\sqrt{b^{T}\mathrm{cov}\left(Y,Y\right)b}} \end{array}$$

In this approach, the CCA coefficients are calculated between EEG measurements recorded by channel O_Z_ and all reference time series. The reference series is a set of Fourier series of the specific frequency period signal (the same frequency *f*_*i*_ of the flickering frequency of LEDs) which can be described as below:

(2) $$\begin{array}{l} Y_{f_{i}}=\left\{\begin{array}{c} \begin{array}{c} \sin\left(2\pi f_{i}t\right)\\ \cos\left(2\pi f_{i}t\right) \end{array}\\ \vdots \\ \sin\left(2\pi N_{h}f_{i}t\right)\\ \cos\left(2\pi N_{h}f_{i}t\right) \end{array}\right\} \end{array}$$

Where *N*_*h*_ = 2 represents the number of harmonics. The frequency with the largest coefficient corresponds to the one of SSVEP (Lin et al., 2006; Duan et al., 2015).

### Motor Imagery

EEG signals from 10 electrodes (CP1, CP2, FC1, FC2, FC3, FC4, C1, C2, C3, C4), which were distributed symmetrically over two hemispheres of the sensorimotor area, were calculated to extract MI features using an improved common spatial pattern method called the complete information common spatial pattern (CICSP). The aim of the conventional CSP is to construct an optimal spatial filter which maximizes the variance of one class while minimizing that of the other using the simultaneous diagonalization of two covariance matrices mathematically (Ramoser et al., 2000). Normally, only a few first and a few last vectors of the spatial filters are most suitable for discrimination of the two MI tasks, which were used for the construction of the classifier, while the CICSP also extracted useful information of the intermediate columns of the spatial filter vector using a dimension reduction method. The EEG signals were firstly bandpass filtered restricting to 9–12 Hz, which encompassed the mu frequency band, and were subsequently spatially filtered with a common average reference (CAR) filter (McFarland et al., 1997). The spatial filter was then calculated by using the CICSP method. Two populations X_1_ and X_2_ related to left and right motor imagery EEG measurements were spatially filtered by Q_1_ and Q_2_, leading to a new time series *Z*_*ij*_, formulated as follows:

(3) $$\begin{array}{l} Z_{ij}=Q_{i}X_{j}\;i,j=\left\{1,2\right\} \end{array}$$

Where i and j denote the index of spatial filters and EEG populations, respectively. Q_1_ and Q_2_ are defined as the m first and the m last column as well as the intermediate column of the spatial filter vector, respectively. The CSP features are calculated by

(4) $$\begin{array}{l} \lambda_{ij}=\text{log}\frac{\text{diag}\left(Z_{\text{ij}}Z_{ij}^{T}\right)}{\text{tr}\left[Z_{\text{ij}}Z_{ij}^{T}\right]} \end{array}$$

Where diag() is the diagonal element of the matrix, tr[·] is the sum of the diagonal elements, *f*_1_ = [λ_11_; λ_12_] represents the first m and last m feature vectors, and *f*_2_ = [λ_21_; λ_22_] represents the intermediate feature vectors of two tasks, and then the dimension canbe reduced by using principal component analysis (PCA) to extract the most useful information, leading to ${f_{2}}^{\prime }$.

(5) $$\begin{array}{l} F=\left[f_{1},{f_{2}}^{\prime }\right] \end{array}$$

*f*_1_ and ${f_{2}}^{\prime }$ were concatenated together to become the complete information feature vectors F, which have been classified using a linear kernel-based support vector machine (SVM) where the SVM classifier model was trained among the calibration dataset (Chang and Lin, 2011).

In addition, during the online implementation, the 1.5-s time window EEG measurement *X*′ was separated into a 0.5-s time window EEG series ${X_{k}}^{\prime }$, where *k* = {1, 2, 3}. ${X_{k}}^{\prime }$ were projected through the intermediate spatial filter *Q*_2_, which was calculated in the calibration phase, then the CSP features ${\lambda_{2k}}^{\prime }$ were calculated by (4). ${\lambda_{2}}^{\prime }=\left[{\lambda_{21}}^{\prime };{\lambda_{22}}^{\prime };{\lambda_{23}}^{\prime }\right]$ were reduced to one dimension, which was considered to be the intermediate feature vector for one EEG measurement.

### Performance Analysis

The analogous information transfer rate (ITR) metric was created for the physical world BCI task.

(6) $$\begin{array}{l} \text{analogous ITR}=\frac{{\text{log}}_{2}(\frac{\text{distance between initial postion to target}}{\text{width of gate}}+1)}{\text{time to pass through gate}} \end{array}$$

The numerator of formula (6) is an index of difficulty computed using the Fitt's law formalization (Decety and Jeannerod, 1995). The displacement from the initial position to the center of the gate for two flight tasks are both 4.75 m, and the width of the gate, as shown in Figure 2B, is 3.5 m. The quadcopter passing through the gate and landing at the designated area was considered as successful completion, whereas flying around the gate or flying beyond the boundaries was deemed to be a failure. It is a relatively simple computation which only related to two distances and emphasizes the ability of the subject who could give specific instructions throughout the entire controlling process, including the time used to correct the instructions (which was unintentional on the part of the subject). This metric is a rough estimate of ITR, and some specific metrics to evaluate the performance of the BCI system were also introduced. The average gate acquisition time (AGAT) was used to evaluate the speed of control, calculated as the total flight time divided by the total number passing through the gate. Out Boundaries Per Unit Time (OBUT) reports the average number of boundary crossings that occurred in each trial. The Percent Task Correct (PTC) metric reports the success rate of the BCI system, which is defined as the number of passes through the gate divided by the sum of the number of passes through gate and failures. The formulas are listed below (Doud et al., 2011).

(7) $$\begin{array}{l} \text{AGAT}=\frac{\text{total flight time}}{\text{numbers of passing through gate }} \end{array}$$

(8) $$\begin{array}{l} \text{OBUT}=\frac{\text{numbers of flying beyond boundaries}}{\text{total flight time}/\text{averaged trial time}} \end{array}$$

(9) $$\begin{array}{l} \text{PTC}=\frac{\text{numbers of passing through gate}}{\left(\text{numbers of passing through gate}+\text{numbers of failure}\right)} \end{array}$$

## Results

### Results in Calibration Phase

The success rate of the three modes was obtained using the whole EEG samples in calibration phase for nine subjects, which is listed in Table 1. According to the results, the average correct recognition rate of SSVEP was 83.44%, the average correct recognition rate of MI was 80.45%, and that of eye blinking was 99.07%. The success rate of the entire task reached 87.65%. The best performance was 97.76% whereas the worst performance was 50.63% in the SSVEP experiment. Similarly, in the detection of MI, the best success rate was 93.70%, and the worst performance was below the level of chance. These two worst success rates came from the same subject (subject 4), who demonstrated to be incapable of the BCI approach. For comparison purposes, Table 2 also shows the performance of the CSP method in MI recognition. It indicated that the CICSP method clearly outperformed CSP among eight of the nine subjects, with an average improvement of 4.45%. A paired *t*-test revealed a significant difference between the accuracy rate for CICSP and CSP (*P* = 0.009). All nine subjects performed remarkably in eye blinking, six of whom reached 100%. Five subjects (subjects 5, 6, 7, 8, and 9) performed at least an 80% success rate for each mode in which they participated in the experiment. The average success rates of SSVEP, MI, and eye blinking among these five subjects were 92.06, 90.20, and 98.75%, respectively. The success rate of the entire task was up to 93.67% among these five subjects.

**Table 2:** Experimental results in terms of success rate for 9 subjects in calibration phase.

**Subject**		**Correct recognition rate**			**Success rate of the entire task**
	**SSVEP**	**MI**		**Eye blinking**	
		**CSP**	**CICSP**		
1	78.44%	82.61%	84.43%	100%	87.62%
2	62.19%	74.72%	78.82%	97.92%	79.64%
3	99.38%	51.54%	60.14%	100%	86.51%
4	50.63%	41.62%	49.63%	100%	66.75%
5	97.76%	86.94%	88.76%	97.92%	94.81%
6	97.06%	91.51%	93.70%	100%	96.92%
7	97.62%	92.04%	91.63%	100%	96.42%
8	87.05%	85.99%	87.97%	95.83%	90.28%
9	80.80%	86.20%	88.95%	100%	89.92%
Average	83.44%	77.02%	80.45%	99.07%	87.65%

*SSVEP, steady-state visually evoked potential; MI, motor imagery*.

### Results in Actual Environment

The performance of the quadcopter flight experiment using the multi-modal BCI system is presented as below. As shown in Table 3, the simple flight task consisted of 10 trials, and two of the five subjects successfully completed PTC with 100% accuracy, and the average for PTC was 92%. Only two subjects navigated the quadcopter beyond boundaries once, and the total flight time was 4.6 min on average. The simple flight task aimed to give subjects a glimpse of the brain-controlled quadcopter, and the experiment results demonstrated that subjects had the ability to reach the gate in succession.

**Table 3:** Experimental results and performance of simple flight task.

**BCI**	**Total trials**	**Numbers of passing through gate**	**Numbers of beyond boundaries**	**PTC (%)**	**Total flight time**
Sub5	10	9	0	90	4.8
Sub6	10	10	0	100	3.8
Sub7	10	10	0	100	4.2
Sub8	10	9	1	90	5.2
Sub9	10	8	1	80	5.1
Average	10	9.2	0.4	92	4.6

*BCI, brain computer interface; PTC, percent task correct*.

In order to provide a comparison between the BCI control and the common approach of control as well as the baseline level, two other experiments were also performed by using a remote controller in absence of the subjects' intent instead of a BCI. In the remote controller experiment, an experimenter who had experience but did not achieve proficiency completed the complex task. In this protocol, the maximum rise and fall speeds were equal to those of BCI. For the horizontal direction, the motion could be in any direction such that not only the two actuations occurred, and the maximum speed was also restricted to that in the BCI control. In addition, the baseline level aimed to identify the extent to which the subjects' performance could be attributed to the success of the BCI system. A subject was instructed to sit quietly and to watch a video of a quadcopter flight in the experimental site, which was considered as fake feedback, without executing the mental or visual task or any eye movement. The BCI system was set up identically to the actual experimental protocol. EEG signals were recorded and controlled the actuation of quadcopter after recognition. It should be noted that poor performance cannot be attributed to the selection of an unacceptable magnitude of EEG or random noise, due to the fact that true EEG signals were employed as the input to the system.

In the complex task, subjects passing through two gates sequentially was considered a success, while passing through one gate was regarded as a half success. Subjects were successful in achieving accurate control of the quadcopter in the actual environment. The performances in various metrics are listed in Table 4 according to the experimental record, and the results of “remote control” and “baseline level” were also evaluated for the purpose of comparison. PTC represented the success rate among all trials. The average among five subjects for PTC was up to 86.5%, which was slightly lower than the RC control (100%) but considerably higher than the baseline level (2.5%). In other words, subjects were able to achieve an average of ~35 gates per 20 trials. Subject 6 reached the highest PTC (97.5%), where 39 gates were hit. Subject 9 passed through 27 gates and performed worst in the calibration phase among the five subjects. It took 26.8 min of total flight time on average for the 20 trials. The best performer, subject 6, spent the shortest amount of time (20.7 min) to complete the flight task, whereas the total flight time of subject 9 was 1.6 times longer than that of subject 6. Because more time was needed to correct the misclassification of commands, the subject had a strong sense of frustration after navigating beyond the boundary, which affected the performance in the following trial. The average time for the quadcopter traveling through a gate was 0.80 min with a constant forward speed of 0.25 m/s, which was nearly 2.5 times longer than the remote-control protocol (0.32 min); however, this was far below the time required for the baseline (20min). The average number of beyond-boundary flights was around 2; thus, the OBUT metric for the BCI control was equal to 0.06, which was far below the baseline. In fact, the quadcopter flew out of the boundary quickly after taking off in each trial. Five subjects who participated in the complex flight experiment displayed an average analogous ITR of 1.69 bit/min compared to 3.69 bit/min of RC protocol, and individual subject values are listed in Table 4. It is a fact that the analogous ITR for the BCI control was 18.8 times higher than that of the baseline level, which is an indication that subjects made an effort to intentionally modulate the EEG signal in order to complete the task at a high success rate.

**Table 4:** Experimental results and performance in various metrics of complex flight task.

	**Total trials**	**Number of successes**	**Number of half successes**	**Number of times passing through gate**	**Numbers of times beyond boundaries**
Sub5	20	16	4	36	0
Sub6	20	19	1	39	0
Sub7	20	19	0	38	1
Sub8	20	16	1	33	3
Sub9	20	12	3	27	5
	**Total flight time (min)**	**AGAT (min/gate)**	**OBUT (Numbers/min)**	**PTC (%)**	**Analogous ITR (bit/min)**
Sub5	27.4	0.76	0	90.0	1.63
Sub6	20.7	0.53	0	97.5	2.33
Sub7	22.1	0.58	0.05	95.0	2.13
Sub8	30.0	0.90	0.10	82.5	1.37
Sub9	34.0	1.25	0.15	67.5	1.00
Average	26.8	0.80	0.06	86.5	1.69
Remote control	–	0.32	0	100	3.90
Baseline	–	20	7	2.5	0.09

*AGAT, average gate acquisition time; OBUT, out boundaries per unit time; PTC, percent task correct; Analogous ITR, analogous information transfer rate*.

## Discussion

The present work demonstrates the capacity for subjects controlling a quadcopter in the real world by a multi-modal EEG-based BCI. The purpose of multi-modal BCI is to increase the number of control commands, reducing the task burden and improving the recognition success rate (Hong and Khan, 2017). It was observed that through this practical BCI, the user could successfully and efficiently navigate a quadcopter to approach a target while avoiding the obstacles in an outdoor actual environment according to a fixed view. The multi-modal BCI system could restore the capacity to explore the real world for disabled people as well as extend the ability of fully capable people in more practical ways. In the future, this multi-modal BCI system could provide more practical purposes. (1) Injured soldiers could rely on it remain in combat in battle fields. (2) It could assist astronauts to accomplish multitask missions in space. (3) Patients with severe disabilities could use them for transporting objects (Nourmohammadi et al., 2018).

We would like to remark that the usage of blinking to facilitate multi-modal BCI is the result of our careful consideration. At the very beginning, the transformation of two modes was completed automatically. If the coefficient in the detection of SSVEPs was higher than a threshold, the EEG block was considered as the SSVEP mode—otherwise, it was considered as the motor imagery mode. Since the detection of the two modes owed to the channels located in two separate areas of the brain, the evoked potential, and the induced potential would not affect each other. Under this circumstance, the LEDs had to keep flickering during the whole process of the experiment. However, gazing at the flickering lights would distract the concentration required for the mental tasks, which led to the increasing number of flights beyond boundaries. Therefore, a switch was introduced in our work to turn on/off the LEDs. Considering the fact that navigating a quadcopter outdoors at a lower altitude is a time-sensitive task, some prototypes have incorporated non-mental features such as eye blinking and muscle movement as a precise switch to minimize the manipulation delay. Since the detection of facial muscle movement needs more electrodes, eye blinking was selected in our work. Once a subject's conscious eye blinking is well-trained with a fixed frequency and the same strength (at least higher than the trained strength), the parameter *d* (the average distance between two troughs) in the blink-recognizing algorithm would remain constant. The parameter *h* (the minimum absolute height of the trough) needs to be estimated using the dataset recorded in calibration phase. Therefore, the blink-recognizing algorithm is robust, and the rest of the BCI system worked properly as well. The quadcopter remains hovering instead of engaging in reckless movement in the case of eye blinking as it does not include mental information.

During the initial phase of the actual quadcopter controlling experiments, the noises generated by the flying quadcopter in the flight field caused the nervousness of some of the subjects to affect the experimental results. Subjects adapted to the environment over time. Since subjects could clearly see the consequence of the task failure such as flying into the bushes, hitting obstacles, and even crashing, this could influence the motivation of the subjects' success in the experiment.

The control commands generated by our current multi-modal BCI system could be further extended. Since CICSP has been demonstrated as an efficient method for multi-class motor imagery classification, in future work we plan to extend the CI-CSP method for multi-class ERD/ERS classification as well as place more flickering LEDs on the screen for generating SSVEP features to actuate more motions on three dimensions.

Generally speaking, the multi-modal EEG signals could be used together to generate a series of control commands for subjects interacting with the real world. The performance of our multi-modal BCI system presents some advantages compared with previous studies (LaFleur et al., 2013; Duan et al., 2015; Wang et al., 2018). Duan et al. utilized a hybrid BCI system on an actual humanoid robot. In the simulation experiment, the average success rate for the entire task was higher than 80%, which was lower than that of our system (93.67%). The overall success rate on an actual device was 73.3%, lower than that for PTC (86.5%), which represented the success rate among all trials we obtained. The manipulation task was achieved with the aid of a visual servo module on a service robot. On the contrary, the quadcopter was controlled using only EEG signals throughout the flight in our experiment. Wang et al. used a 4-class SSVEP which was presented in a virtual reality environment to control a simulated quadcopter. The online accuracy achieved 78%, which was lower than the overall accuracy rate of our multi-modal BCI (86.5%). Although it alleviates the user's visual burden to some extent, it is only conducted in the simulated environment. LaFleur et al. conducted a quadcopter control in the physical word using unitary MI mode. The average ITR of this work was 1.16, which was lower than that of our multi-modal BCI system (1.69). The results indicate that our multi-modal BCI system could effectively increase the accuracy rate while alleviating both mental burden and visual fatigue to a large extent.

## Conclusion

This paper presents a multi-modal BCI system to accurately and stably control a quadcopter to pass through gates in three-dimensional physical space. The MI and SSVEP modes—which are associated with two types of regular EEG patterns, ERD/ERS, and SSVEP—were employed to actuate the flight on both horizontal and vertical directions. Two modes were rapidly switched by eye blinking. The ERD/ERS and SSVEP patterns were analyzed by using the CICSP feature extraction method and the CCA frequency recognition method, respectively. Eye blinking was detected by counting the peak method within each EEG data block. Subjects were able to navigate the quadcopter passing through a series of gates in the outdoor environment continuously, accurately and rapidly. Several metrics for real-world BCI systems were used to assess the performance of this system. The PTC reached 86.5% and the analogous ITR attained 1.69 bit/min for five subjects, with the average gate acquisition time being nearly 0.80 min. This system could build the ability for people who suffer from paralyzing disorders to interact with three-dimensional real world. The multi-modal BCI could increase the accuracy rate while alleviating both the mental burden and the visual fatigue to a large extent.

## Ethics Statement

This study was carried out in accordance with the recommendations of guiding research into human subjects, Northwestern Polytechnical University Hospital Ethics Committee with written informed consent from all subjects. All subjects gave written informed consent in accordance with the Declaration of Helsinki. The protocol was approved by the Northwestern Polytechnical University Hospital Ethics Committee.

## Author Contributions

XD, SX, and XX contributed equally to this work. They conceptualized the idea and proposed the experimental design. XD also programmed and performed all data analysis as well as wrote the manuscript at all stages. SX and XX revised the manuscript at all stages. YM and ZX conducted the experiments. All authors read and approved the final manuscript.

### Conflict of Interest Statement

The authors declare that the research was conducted in the absence of any commercial or financial relationships that could be construed as a potential conflict of interest.
